# Synthesis and characterization of sustainable Zinc Oxide nanoparticles for waxy crude flow assurance application

**DOI:** 10.1371/journal.pone.0358989

**Published:** 2026-09-28

**Authors:** Abubakar Aji, Hisham Khaled Ben Mahmud, Abdullah Abduljabbar, Ibnelwaleed A. Hussein, Nik Ahmad Nizam Nik Malek, Victor Damilare Ola, Mysara Eissa Mohyaldinn

**Affiliations:** 1 Department of Petroleum Engineering, Universiti Teknologi PETRONAS (UTP), Seri Iskandar, Perak Darul Ridzuan, Malaysia; 2 Faculty of Engineering, Sohar University, Oman; 3 Center of Flow Assurance, Institute of Sustainable Energy & Resources, Universiti Teknologi PETRONAS (UTP), Seri Iskandar, Perak Darul Ridzuan, Malaysia; 4 Gas Processing Center, College of Engineering, Qatar University, Doha, Qatar; 5 Chemical Engineering Department, College of Engineering, Qatar University, Doha, Qatar; 6 Centre for Sustainable Nanomaterials (CSNano), Ibnu Sina Institute for Scientific and Industrial Research (ISI-ISIR), Universiti Teknologi Malaysia (UTM), Skudai, Johor, Malaysia; Wadi International University, SYRIAN ARAB REPUBLIC

## Abstract

Wax precipitation in crude oil pipelines is a challenge to production. Current inhibitors raise concerns about toxicity and environmental impact. Hence, the need for sustainable alternatives arises. This study aims to explore the *Vitex doniana*-assisted synthesis, characterization, and application of zinc oxide nanoparticles as crude oil wax inhibitors. The nanomaterial was characterized using Fourier Transform Infrared Spectroscopy, Gas Chromatography–Mass Spectrometry, X-Ray Diffraction, Field Emission Scanning Electron Microscopy, Energy-Dispersive X-Ray Spectroscopy, and Thermogravimetric Analysis. The application of 0.04 wt% zinc oxide was evaluated based on wax appearance temperature, rheological properties, and wax-inhibition efficiency. Fourier Transform Infrared Spectroscopy analysis on the ethanolic *Vitex doniana* leaf extract confirmed the presence of alcohols, phenolics, carbonyls, aromatics, proteins, and polyphenols responsible for nanoparticle reduction and stabilization of the nanoparticle. Gas Chromatography–Mass Spectrometry of the extracts identified 18 distinct peaks in the organic profile, revealing a mixture of esters, aldehydes, acids, and heterocycles. The dominance of n‑hexane (34.9%) and glycidyl oleate (21.2%) highlights hydrocarbon and ester fractions as major components. X-Ray Diffraction analysis confirmed zinc oxide nanocrystals in a pure hexagonal wurtzite phase (a = 3.243 Å, c = 5.195 Å) with an average crystallite size of 36.1 nm and microstrain of 1.4 × 10 ^−^ ⁴, evidencing high crystallinity and suitability for optoelectronic and photocatalytic applications. Field Emission Scanning Electron Microscopy-Energy-Dispersive X-Ray Spectroscopy confirmed elemental composition of 64.3 wt% Zn, 20.6 wt% O, 15.1 wt% residual C, and trace N, evidencing high purity. Thermogravimetric Analysis indicated high thermal stability with minimal mass loss (1.308% at ~118.7 °C, 3.340% at ~227.9 °C, and 7.427% at ~391.8 °C; total mass loss < 12.1% up to ~800 °C). Rheological analysis showed a reduction in viscosity of up to 56.2% at 23 °C when crude oil was blended with zinc oxide nanoparticles. The Cross-Polarized Microscopy test showed that zinc oxide modified the wax structure, resulting in clustered morphology, suggesting a cocrystallization mechanism. The Arrhenius plot of viscosity showed that zinc oxide reduced the flow activation energy from 213.6 kJ mol ^−^ ¹ to 207.6 kJ mol ^−^ ¹, confirming the effectiveness of zinc oxide nanoparticles in improving crude oil flow by disrupting wax-crystal networks. These findings confirm the successful production of high-quality, thermally stable, and surface-functionalized zinc oxide nanoparticles, suitable for various industrial applications, especially waxy crude flow assurance.

## Introduction

Wax precipitation in crude oil pipelines presents significant flow assurance challenges, increasing operational expenses and production risks [1]. Waxy crude oils are prone to solidification under sub-ambient pipeline conditions, leading to wax deposits that restrict flow, reduce pipeline efficiency, and increase operational costs [2]. Conventional wax mitigation techniques, such as chemical pour point depressants and thermal or mechanical methods, are widely employed. However, they have several limitations, including high cost, energy-intensive operation, and potential environmental hazards associated with chemical use [3]. These drawbacks necessitate the development of alternative, environmentally benign materials that mitigate wax precipitation and deposition in a more sustainable manner.

In recent years, the escalating demand for sustainable and environmentally benign technologies has propelled the field of green synthesis to the forefront of materials science [4]. This approach emphasizes the use of natural resources and eco-friendly methods for material production, minimizing reliance on hazardous chemicals typically associated with conventional synthesis routes [5,6]. On the other hand, nanomaterials, particularly metal oxide nanoparticles (NPs) such as Zinc oxide (ZnO), have attracted significant attention due to their diverse applications including optoelectronics, photocatalysis [7], electrochemical performance [8] and medicine [9]. However, traditional methods for synthesizing the NPs often involve toxic precursors and generate harmful byproducts, necessitating the exploration of cleaner alternatives [10].

Green synthesis is an environmentally friendly approach to synthesizing materials, including nanomaterials, using sustainable methods and minimizing hazardous chemicals. It involves using natural resources, such as plant extracts, microorganisms, and enzymes, as reducing and capping agents in the synthesis process. Among the various green synthesis strategies, the use of plant extracts has emerged as a promising approach. This biological mediation offers a cost-effective, scalable, and non-toxic route for nanoparticle (NP) fabrication [11]. Specifically, plants contain phytochemicals such as flavonoids, alkaloids, and terpenoids, which can act as reducing and stabilizing agents, and could be used in the synthesis of metal oxide nanocomposites, while the resulting nanomaterial can serve as an environmentally benign wax inhibitor, aligning with the goals of sustainable flow assurance [12].

This study focuses on the green synthesis of ZnO NPs utilizing the leaves extract of *Vitex doniana (V. doniana)*, as well as characterization, and application of the nanoparticles for crude oil wax inhibition. The choice of this plant is motivated by its traditional medicinal uses and the presence of bioactive compounds that can effectively mediate the synthesis process [13].

The *V. doniana* (Fig 1, SD1) is widely distributed in Africa, particularly in Ghana, Burkina Faso, Nigeria, and Cameroon [14]. It produces small, white flowers and spherical, green fruits that turn black-purplish when ripe. The fruits have a woody seed, an approximate height of 1.5−20 cm, and a width of 1.0–1.2 cm. The raw fruits are edible [15].

**Fig 1:**
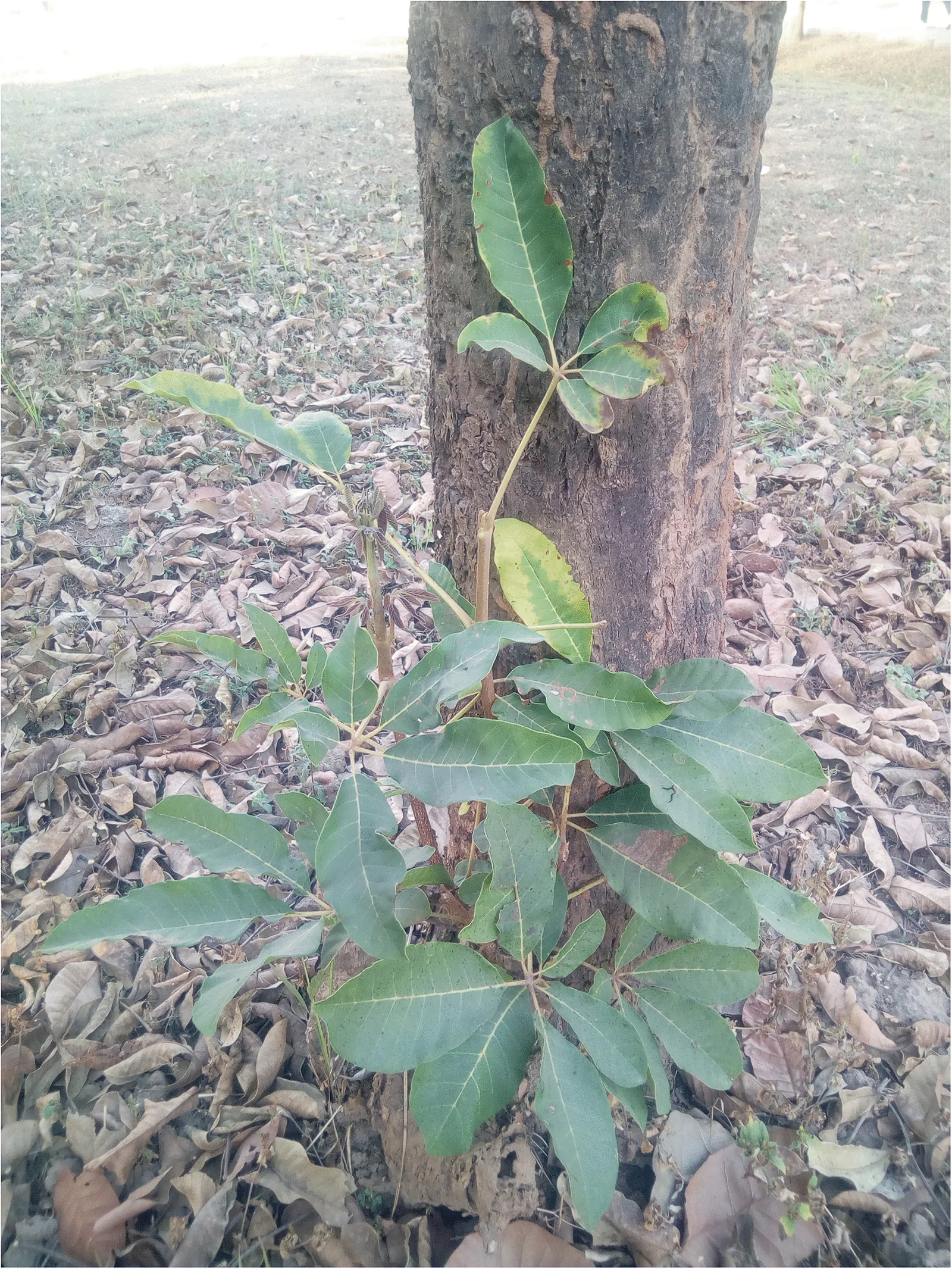
*V. doniana* Tree trunk and leaves.

The plant is known in English, Hausa, Fulani, and Yoruba as Black plum, Dinya, Galbilhi, Oori-nla, respectively [16]. It is a widely used multipurpose plant in tropical Africa [17], valued for its nutritional, medicinal, and economic significance (Table 1, T1).

**Table 1:** Traditional and Scientific Importance of V. doniana for Green Synthesis of ZnO NPs.

Name	English (black plum, Vitex, African Oak), Fula (Galbihi), Hausa (Dinya), Fulani (Galbilhi), Kanuri (Ngarmi), Yoruba (Oori-nla), and in Nupe it is called Dinchi/Edin [16]
Leave ingredient (composition)	Flavonoids, steroids, phlobatannins, alkaloids, saponins, phenolic acids, tannins, terpenoids [18]. Vitamins A, C, and E, as well as minerals like iron and copper [19]
Targeted part	Leave (aqueous and methanolic extracts)
Example of applications	In traditional medicine, it is used to treat anemia, diarrhea, and liver disorders. Pharmacological activities include antioxidant, anti-inflammatory, antimicrobial, antidiabetic, hypolipidemic, hepatoprotective, and anticancer effects [20]
Useful functional groups	Hydroxyl (-OH), carbonyl (C = O), amine (-NH2), and phenolic groups, which facilitate reduction and stabilization of metal ions during NP synthesis [21].
Motivation behind selecting the plant	-Abundance and easy accessibility in tropical Africa. - Rich phytochemical profile with strong reducing and capping potential. - Eco-friendly, cost-effective, and sustainable alternative for NP synthesis. - No published evidence was found in the literature regarding the application of *V. doniana* in ZnO NPs green synthesis

Green synthesis has rapidly evolved, with increasing research exploring diverse botanical precursors for metal-oxide nanoparticle fabrication. However, comprehensive systematic literature reviews in the fields of sustainable wax mitigation [22], and green synthesis methods [23] reveal that while various plant species have been utilized for nanoparticle synthesis, *V. doniana* remains significantly under-explored. This underscores a significant research gap at the intersection of sustainable nanotechnology and petroleum flow assurance. Therefore, the present study seeks to (i) synthesize ZnO NPs using the extracts of *V. doniana* leaves, (ii) characterize the properties of the biogenic NPs, and (iii) evaluate their efficacy in inhibiting paraffin wax so as to improve the rheological behavior of waxy crude oils.

## Methodology

The plant extract was analyzed using Fourier Transform Infrared Spectroscopy (FTIR) and Gas Chromatography–Mass Spectrometry (GC-MS). At the time, the biosynthesized ZnO NPs were characterized using X-Ray Diffraction (XRD), Field Emission Scanning Electron Microscopy (FESEM), Energy-Dispersive X-Ray Spectroscopy (EDS), and Thermogravimetric Analysis (TGA) to evaluate their functional groups, crystalline structure, thermal stability, morphology, elemental composition, and purity. For quantitative particle size analysis, FESEM micrographs were further processed using a high-throughput Python-based workflow (SciPy, NumPy, and Pillow). For particle sizing, FESEM images were analyzed using a calibrated Python-based workflow with Gaussian normalization and threshold-based segmentation, followed by a distance-transform-based seed-detection algorithm to resolve agglomerated particles. As for the application studies, Cross-Polarized Microscopy (CPM) and rheology tests were also conducted to assess the efficacy of the NPs on waxy crude oil.

## Material and Instrument

The reagents used were Sodium Hydroxide (NaOH), Zinc Nitrate [Zn(NO_3_)_2_], Ethanol (C_2_H_6_O), and the plant extract. The instruments used were a hot plate with mixer, a furnace, a KUBOTA Centrifuge, a Drying Oven, an Ultrasonic Homogenizer, FTIR (Brand: Perkin Elmer, Model: Frontier 01), GC-MS (Brand: Agilent, Model: GC 5877), XRD (Panalytical Model, brand Xpert3 Powder), TGA (Brand: Perkin Elmer, Model: STA6000) and FESEM (Zeiss brand, model V55). As for the software, Origin, and HighScore were used to analyze some of the results.

A hot plate was used for controlled heating and mixing, while the furnace enabled high-temperature calcination for ZnO crystallization. The KUBOTA centrifuge ensured efficient NP separation, and the drying oven facilitated moisture removal. Analytical instruments (FTIR, GC–MS, XRD, and FESEM) were employed for structural, morphological, and compositional analysis, ensuring comprehensive characterization. A rheometer was used for the rheology test.

### Extraction of *V. doniana Leaves*

Fresh leaves of *V. doniana* were collected on 13 May 2024 from the school premises of Nile University of Nigeria, Abuja, Nigeria. This was the late dry‑to‑early rainy season transition, a period known to influence phytochemical composition. Dr. Namla identified the plant from the Department of Biological Sciences, Nile University of Nigeria. The leaves were dried in the shade at room temperature to protect the phytochemicals from degradation by sunlight. Approximately 115 g of fresh *V. doniana* leaves yielded 100 g of leaf powder, based on the reported moisture content of 13.08% [24], giving a fresh‑to‑powder ratio of 1.15: 1. Once dried, the leaves were chopped and ground into a fine powder using a blender for extraction.

The dried and ground leaves (100 g) of *V. doniana* were boiled in deionized water for 30 minutes at 100 °C, continuously stirred at 200 rpm using a magnetic stirrer, to extract the bioactive components. After boiling, the mixture was filtered to obtain the clear leaf extract. The filtrate was stored in the refrigerator for further use in the synthesis process [25].

Accurate FTIR results require a solvent-free sample to avoid solvent-induced peak overlap. Consequently, ethanol was employed to extract the phytochemicals using a Soxhlet extractor [26]. A 10g sample *of V. doniana leaves was* extracted for 6 hours until the color of the recycling solvent became pale, indicating complete extraction. The solvent was subsequently removed from the samples using a rotary evaporator, and the residue was dried in an oven to a constant weight before submission for the analysis [27].

### Synthesis of ZnO NPs

To synthesize ZnO NPs, 10 mL of aqueous extract of *V. doniana* was added to Erlenmeyer flasks and brought up to 50 mL with distilled water. It was then stirred for 10 minutes at 150 rpm at room temperature. A 50 g mass of Zn(NO_3_)_2_ was dissolved in the above extract solution. It was then stirred for 30 minutes at 150 rpm at room temperature. The mixture was incubated in a boiling water bath at 70 °C for 10 min until a brown precipitate formed, then allowed to cool at room temperature under continuous stirring for 15 min. Freshly prepared 0.5 M NaOH solution (60 mL) was added under continuous stirring until the color changed and a milky precipitate formed. Green-synthesized ZNO NPs were purified by centrifugation at 10,000 rpm for 30 min. The final product suspension was dried at room temperature for 5 days, oven-dried at 120 °C for 4 h, and calcined in the furnace at 600 °C [28].

### Characterization of Components

Characterization plays a pivotal role in understanding the physicochemical properties of green-synthesized NPs, as these attributes directly influence their performance in targeted applications such as wax inhibition in crude oil systems. The efficiency of ZnO NPs in preventing paraffin wax precipitation is strongly dependent on their size, surface morphology, crystallinity, thermal stability, and surface functional groups, which collectively determine their dispersion ability, interaction with crude oil constituents, and capacity to modify wax crystallization behavior [29].

A combination of analytical techniques was therefore employed to comprehensively examine the synthesized material. FESEM provides insights into the surface morphology and particle size distribution, which are critical for nanoparticle-to-oil interface interactions. XRD analysis elucidates the crystalline structure and phase purity, parameters essential for correlating structural properties with wax inhibition efficiency. EDS complements this by confirming elemental composition. TGA assesses the thermal stability and decomposition patterns of ZnO NPs to ensure suitability for high-temperature pipeline environments. Meanwhile, FTIR and GC-MS are applied to the *V. doniana* leaf extract to identify phytochemicals and functional groups responsible for the reduction, stabilization, and surface modification of the NPs [30].

Together, these characterization tools provide critical parameters, including particle size, crystallinity, elemental composition, thermal stability, and bioactive functional groups, that establish the foundation for understanding *V. doniana*-mediated ZnO NPs as sustainable wax inhibitors. Fig 2 (SD2) shows the synthesized ZnO NPs.

**Fig 2:**
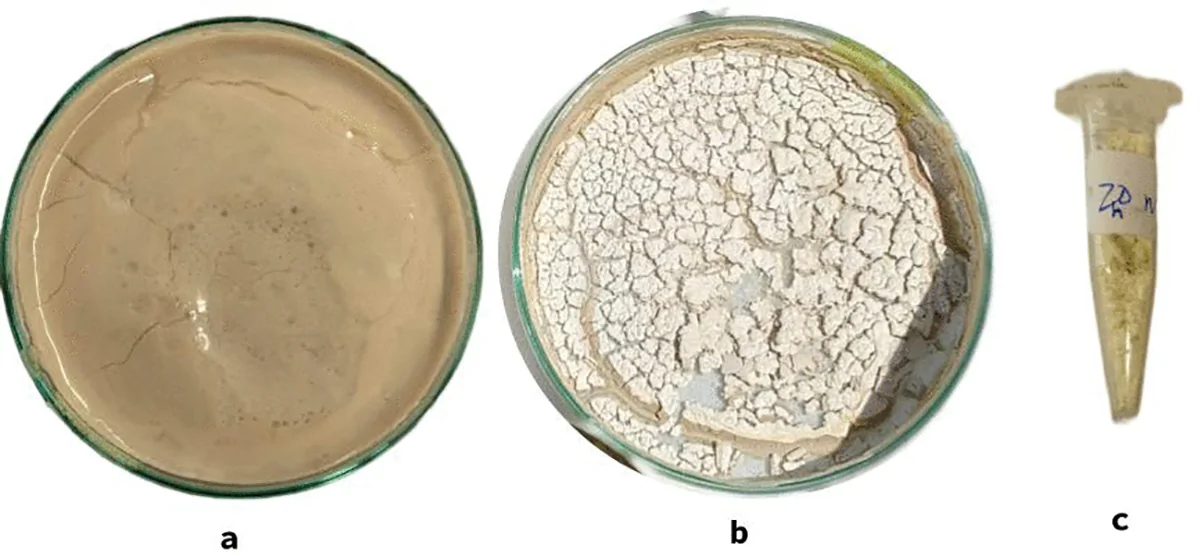
Progressive stages in the synthesis of ZnO NPs. (a) before oven drying, (b) after oven drying, and (c) after calcination.

### Application of ZnO to Crude Oil

Accurate investigation of the interaction between crude oil and inhibitors necessitated proper sample preparation. One hundred grams (100g) of Dulang crude oil were measured into a beaker and subsequently preheated in an oven at 70 °C for 90 minutes (1.5 hours). This preheating step was essential to guarantee the complete dissolution of any wax that had previously crystallized within the oil [31]. Subsequently, 0.04 wt% [39] of the synthesized ZnO NPs were blended with the crude oil using an ultrasonicator [32]. The actual settings to disperse the nanomaterial are power = 500, T-Run = 10 minutes, T-On=5.0 s, T-Off=0.05 s, and temperature = 70 °C. This procedure ensured uniform dispersion of ZnO NPs within the crude matrix, enabling reliable evaluation of their influence on wax inhibition and flow behavior. The subsequent analyses focused on compositional characterization and rheological performance to determine the effectiveness of the nanoparticles.

## Results and discussion

The compositional and rheological analyses were conducted to understand how ZnO NPs interact with the waxy components of Dulang crude oil. The results provide insight into the crude’s molecular composition, wax content, and flow characteristics, forming the basis for evaluating the nanoparticle’s inhibition efficiency.

### Composition of Dulang Crude Oil

The chemical composition and key physical properties of Dulang crude oil were compiled from experimental determinations in this work alongside established literature values; Table 2 (T2). Specifically, the hydrocarbon composition and refractive index were measured in this study, whereas physical properties such as specific gravity, density, API gravity, and molecular weight were retrieved from previous reports [1,33]. Notably, the wax content was reported as 19.50 wt%, which was obtained from the literature [34].

**Table 2:** Dulang crude oil composition.

Entry	Parameter/ Component	Mole Fraction (%)/ Value	Reference
1	3-Methylpentane (C_6_)	4.36	This work
2	n-Hexane (C_6_)	30.84	This work
3	Methylcyclopentane (C_6_)	5.66	This work
4	Undecane (C_11_)	0.95	This work
5	Dodecane (C_12_)	1.33	This work
6	Tridecane (C_13_)	1.74	This work
7	Tetradecane (C_14_)	2.14	This work
8	Pentadecane (C_15_)	2.23	This work
9	Heptadecane/Heneicosane (C_17_–C_21_)	30.42	This work
10	Docosane (C_22_)	2.73	This work
11	Tetracosane (C_24_)	4.80	This work
12	Pentacosane (C_25_)	2.77	This work
13	Hexacosane (C_26_)	2.70	This work
14	Heptacosane (C_27_)	2.60	This work
15	Triacontane (C_30_)	1.41	This work
16	Total Hydrocarbon Fraction	100.00%	This work
17	Refractive Index	1.47	This work
18	Specific Gravity	0.8378	[1]
19	Density (g cm ^−^ ³)	0.837	[1]
20	API Gravity (°API)	37.39	[1]
21	Wax Content (wt%)	19.50	[34]
22	Molecular Weight (kg kmol ^−^ ¹)	189.85	[33]

The crude exhibits a specific gravity of 0.8378 and a density of 0.837 g cm ^−^ ³, classifying it as a medium crude oil. The relatively high average molecular weight (189.85 kg kmol ^−^ ¹) further reflects the predominance of paraffinic components but the American Petroleum Institute (API) gravity of 37.39 places the crude within the moderately light category, indicating a system with favorable gravity yet substantial wax-forming potential.

From a flow-assurance perspective, the dominance of long-chain hydrocarbons significantly increases the likelihood of wax crystallization at reduced temperatures, leading to elevated viscosity and impaired flow behavior. Such compositional characteristics necessitate effective mitigation strategies, including wax inhibitors, thermal control, or crude blending, to ensure operational reliability. Despite its moderately light API gravity, the high proportion of heavy paraffins and substantial wax content suggests pronounced flow-assurance challenges, making this crude oil particularly suitable for rheological modeling and wax-deposition mitigation studies.

### FTIR Evaluation of *V. doniana* Leaves Extract

The FTIR spectrum of *V. doniana* leaf extract in Fig 3 (SD3) highlights the presence of several bioactive functional groups that are pivotal in the green synthesis of metal oxide NPs like ZnO. These functional groups function as reducing, stabilizing, and capping agents, facilitating NP formation and ensuring colloidal stability.

**Fig 3:**
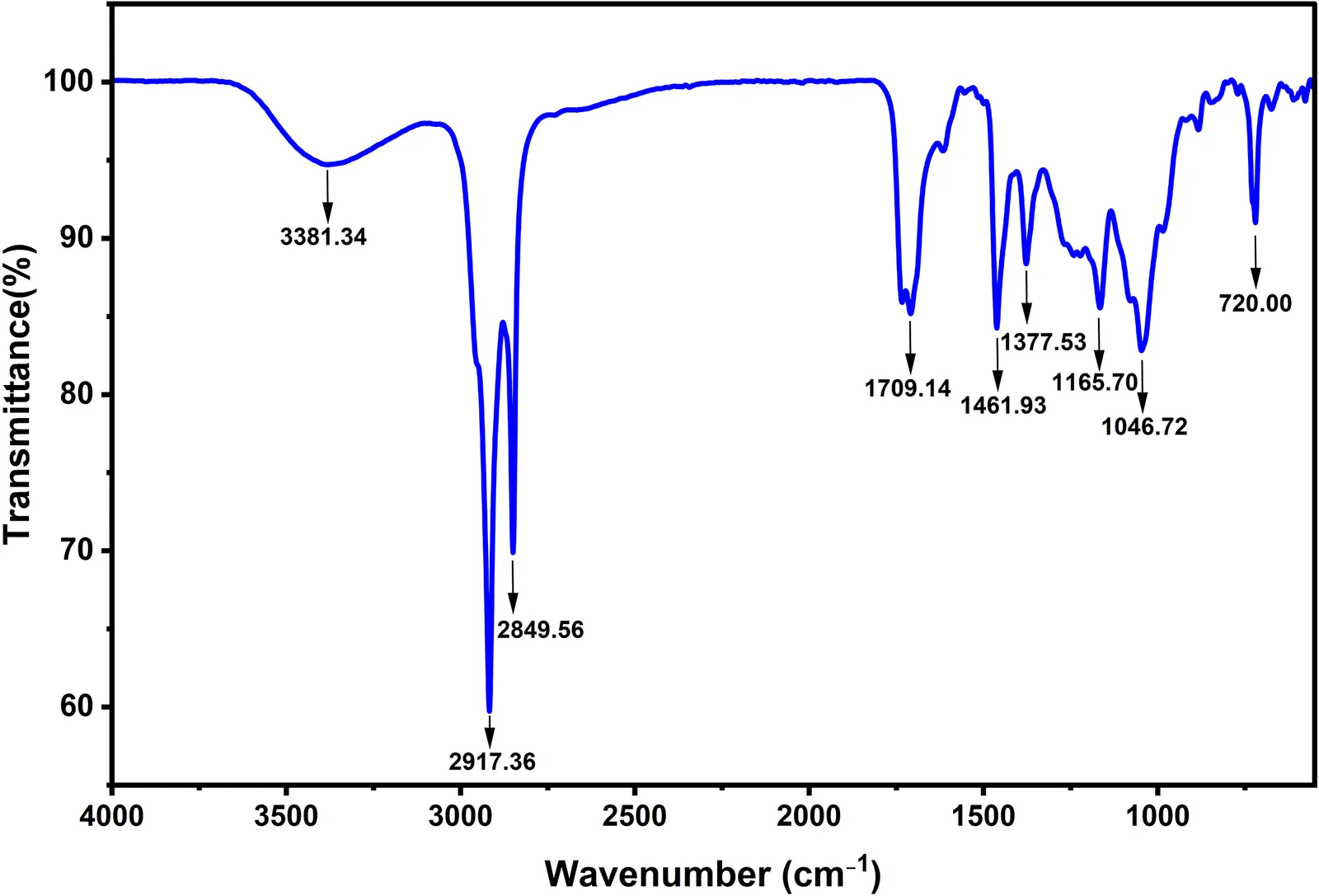
FTIR of *V. doniana* leaf extract illustrating major absorption bands.

The FTIR spectrum of the *V. doniana* leaf extract exhibits several prominent absorption bands that reflect the presence of diverse phytochemical constituents responsible for its reducing and stabilizing abilities in NP synthesis. A broad and weak band observed in the region of 3400–3300 cm ^−^ ¹ corresponds to O–H stretching vibrations, typically associated with alcohols and phenolic compounds [33]. These groups are widely reported in plant extracts and are known to play critical roles in hydrogen bonding and metal ion reduction. A strong and sharp absorption in the 2920–2850 cm ^−^ ¹ region is indicative of C–H stretching of aliphatic −CH_2_ and −CH_3_ groups, confirming the presence of long-chain hydrocarbons and other organic metabolites [35].

A distinct and intense peak around 1730–1700 cm ^−^ ¹ is attributed to the C = O stretching of carbonyl groups, commonly found in aldehydes, ketones, esters, or carboxylic acids [36,37]. The strength of this band suggests a considerable abundance of carbonyl-containing biomolecules, which are often reported as effective capping and stabilizing agents during NP formation. Additionally, absorptions located between 1650 and 1550 cm ^−^ ¹ correspond to C = C stretching vibrations of aromatic rings and, in some cases, amide I and II bands arising from proteins or alkaloids. These compounds further contribute to reduction and stabilization processes [38].

The region between 1500 and 1000 cm ^−^ ¹ displays multiple peaks indicating the presence of C–O, C–N, and C–O–C stretching vibrations, typically associated with polyphenols, flavonoids, glycosides, and other oxygenated phytochemicals [36,37]. The complexity of this fingerprint region reflects the heterogeneous phytochemical profile of *V. doniana*. Hence, the identified functional groups confirm that the extract contains abundant polyphenols, flavonoids, terpenoids, and carbonyl-bearing compounds (Table 3, T3), all of which are known to enhance its capacity as a reducing and stabilizing agent in green synthesis of metal or metal-oxide nanomaterials.

**Table 3:** *V. doniana* functional groups and organic compounds summary.

FTIR Region (cm ^−^ ¹)	Functional Group(s)	Phytochemical Class	Role in NP Synthesis	Citations
3400–3300	O–H (alcohols, phenols)	Polyphenols, flavonoids	Hydrogen bonding, metal ion reduction	[37,39]
2920–2850	C–H (aliphatic –CH_2_/–CH_3_)	Hydrocarbons, terpenoids	Organic matrix, stabilization	[35,39]
1730–1700	C = O (carbonyl: aldehydes, ketones, esters, acids)	Carbonyl compounds	Capping, stabilization, reduction	[36,37]
1650–1550	C = C (aromatic), amide I/II	Aromatics, proteins, alkaloids	Reduction, stabilization	[35,38]
1500–1000	C–O, C–N, C–O–C	Polyphenols, glycosides, flavonoids	Capping, stabilization	[36,37]

Hence, the FTIR identified functional groups summarized in the above table confirm that *V. doniana* leaf extract possesses a rich array of oxygenated and aromatic phytochemicals. This multifunctional composition underscores its suitability as a sustainable bio-reductant for the green synthesis of metal and metal-oxide nanoparticles, such as ZnO NPs.

### GC-MS evaluation of the plant leaves extract

The Total Ion Chromatogram (TIC) of *V. doniana* green-synthesized NP is shown in Fig 4 (SD4). The chromatogram shows a complex chemical profile with 18 distinct peaks spanning retention times from approximately 1.4 to 46.7 minutes. This broad retention range suggests the presence of a diverse mixture of volatile and semi-volatile organic compounds, characteristic of medicinal plant extracts.

**Fig 4:**
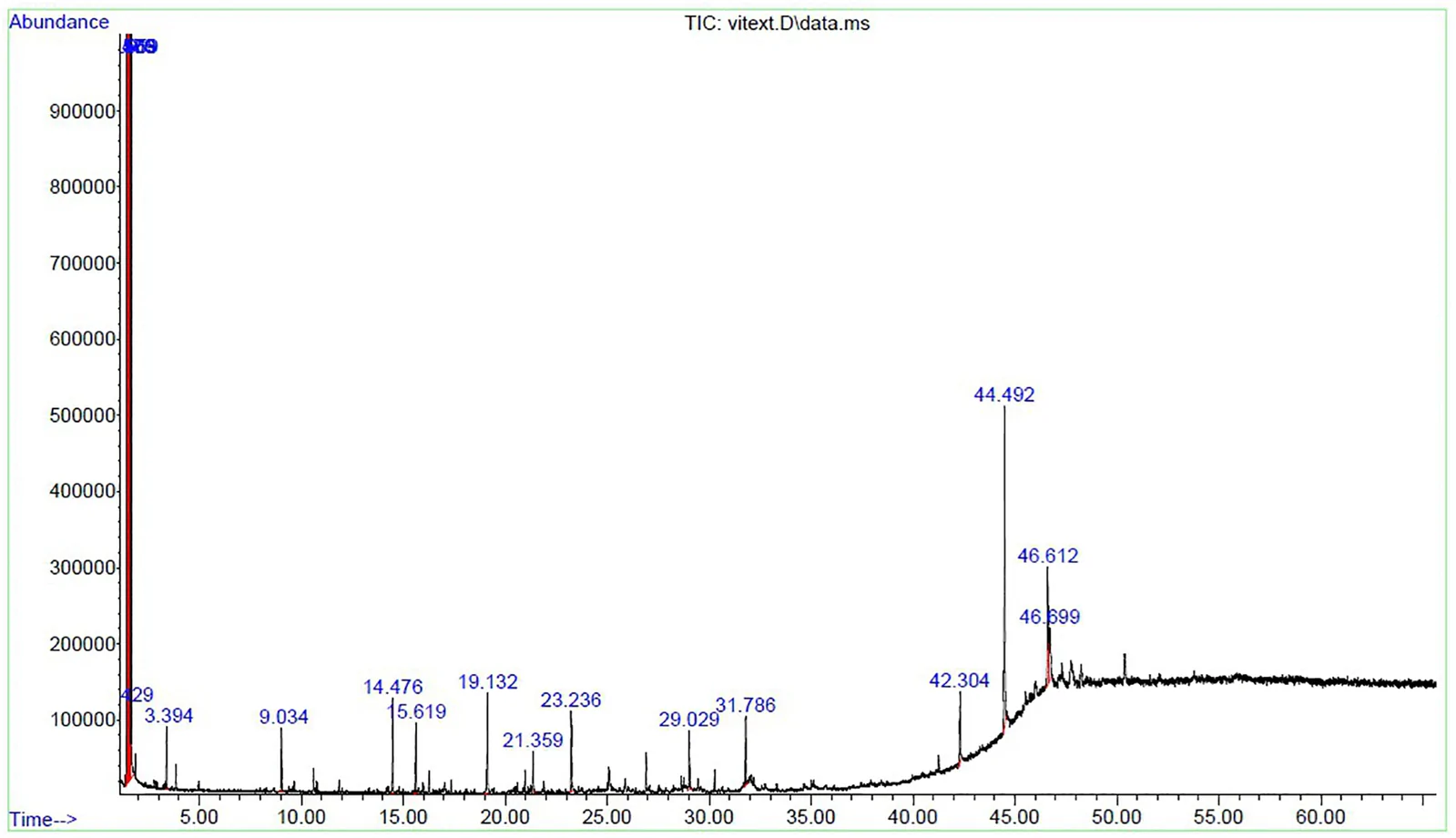
GC-MS analyses showing TIC of *V. doniana* leaf extract.

The GC–MS profile revealed a mixture of hydrocarbons, esters, aldehydes, acids, and heterocycles as shown in Table 4 (T4). Early retention peaks (RT 1.47–1.66 min) corresponded to volatile alkanes such as 3‑methylpentane, n‑hexane, and methylcyclopentane, while later peaks (RT 34–37 min) identified glycidyl esters and long-chain aldehydes, indicating lipid-derived constituents. Oxygenated heterocycles (oxetane, tetrahydrofuran, dioxolane) and dicarboxylic acid esters were also detected, providing functional groups (–OH, –C = O, –C–O–C) relevant for reduction and capping in NP synthesis. The dominance of n‑hexane (34.9%) and glycidyl oleate (21.2%) highlights the hydrocarbon and ester fractions as major components, supporting the role of *V. doniana* phytochemicals in mediating the formation of ZnO NPs.

**Table 4:** GC–MS identified organic compounds from *V. doniana* leaf extract.

Pk No	RT (min)	Area%	Compound Name	Quality
1	1.477	6.89	Pentane, 3-methyl-	90
			Pentane, 3-methyl-	90
			Pentane, 3-methyl-	90
2	1.513	34.92	n-Hexane	86
			2-Ethyl-oxetane	72
			Furan, tetrahydro-3-methyl-	58
3	1.563	13.88	n-Hexane	86
			2-Ethyl-oxetane	78
			Furan, tetrahydro-3-methyl-	58
4	1.661	8.81	Cyclopentane, methyl-	90
			Cyclopentane, methyl-	90
			Cyclopentane, methyl-	86
5	34.701	1.95	Glycidyl palmitate	91
			2-Dodecylcyclobutanone	30
			Adipic acid, 4-heptyl nonyl ester	14
6	37.149	21.16	Glycidyl oleate	99
			9-Octadecenal, (Z)-	94
			13-Octadecenal, (Z)-	49
7	37.294	3.33	Succinic acid, 1-cyclopentylethyl ethyl ester	47
			Adipic acid, butyl cycloheptyl ester	47
			Adipic acid, cyclohexyl isobutyl ester	43
8	37.479	4.03	Glycidyl palmitate	90
			1,15-Pentadecanedioic acid	30
			Myristoyl chloride	30
9	52.119	5.03	1,3-Dioxolane, 4-ethyl-5-octyl-2,2-bis(trifluoromethyl)-, trans-	43
			1-Methyl-4-(1-methylethyl)-3-[1-methyl-1-(4-methylpentyl)-5-methylheptyl]cyclohexene	38
			1,3-Dioxolane, 4-ethyl-5-octyl-2,2-bis(trifluoromethyl)-, cis-	38

The GC–MS results presented in the table above provide a molecular fingerprint of *V. doniana* leaf extract, revealing its complex blend of bioorganic constituents. The coexistence of short-chain volatiles and long-chain lipid derivatives suggests a balanced chemical environment that supports both rapid reaction kinetics and sustained nanoparticle growth, thereby contributing to morphology control and enhanced functional performance [40].

### XRD evaluation of ZnO

The crystalline structure of the synthesized ZnO nanoparticles was examined using XRD, as illustrated in Fig 5 (SD5), to determine the phase composition and microstructural parameters. The sharp and intense peaks observed in the diffractogram indicate a high degree of crystallinity in the ZnO NPs. Notably, no secondary phases or impurity-related peaks were detected, suggesting that the synthesized ZnO is phase-pure.

**Fig 5:**
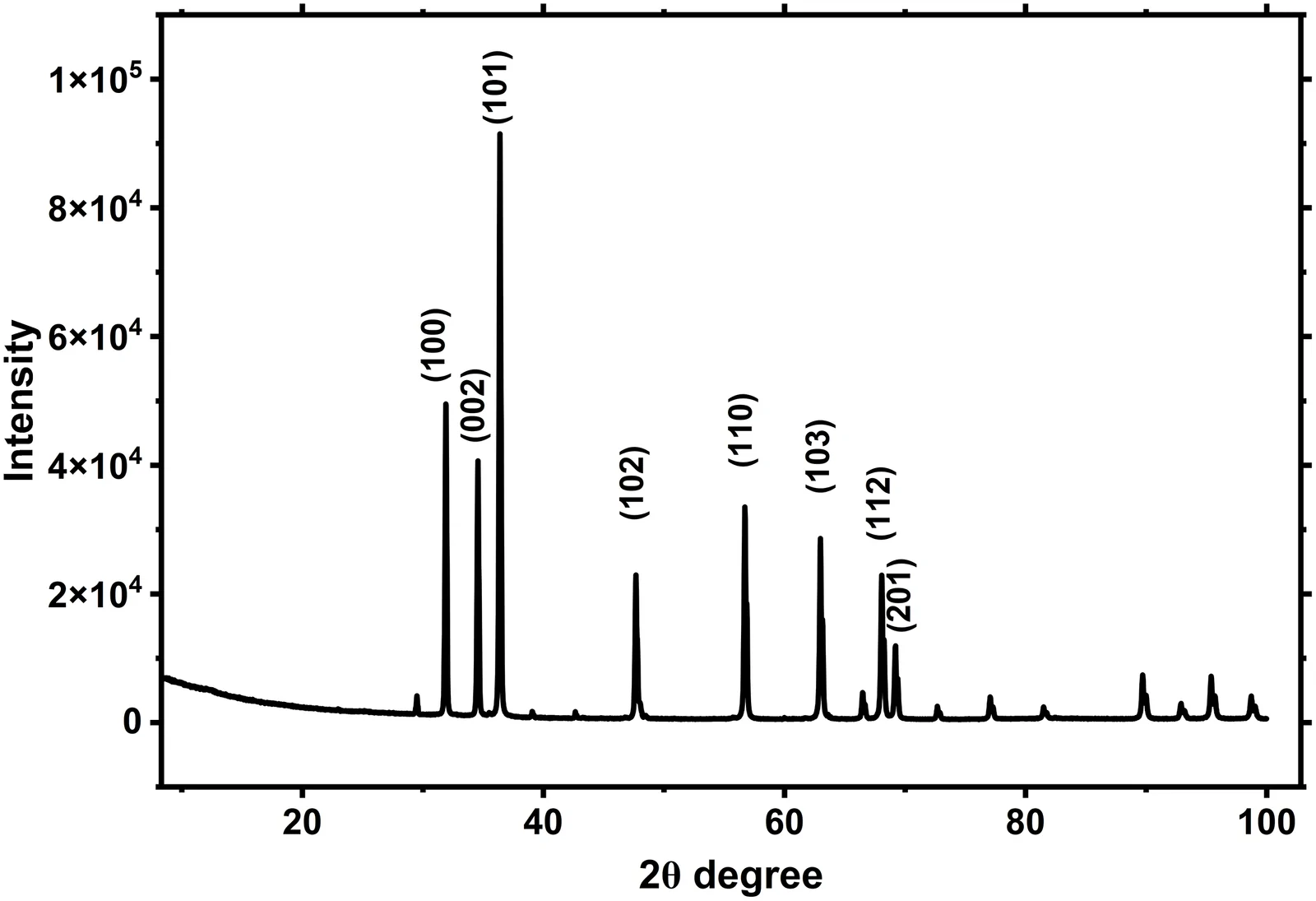
XRD pattern of ZnO NPs showing sharp diffraction characteristic peaks.

The observed diffraction peaks at 2θ = 31.83°, 34.50°, 36.33°, 47.65°, 56.73°, and 63.01° correspond to the (100), (002), (101), (102), (110), and (103) planes of ZnO, respectively. The dominant (101) reflection at 2θ = 36.33° is characteristic of the wurtzite ZnO phase, as confirmed in the literature [41–43].

The diffraction pattern confirmed a single-phase hexagonal wurtzite structure [43] with space group P6_3_mc. The lattice constants a and c for the hexagonal lattice system were determined using the hexagonal interplanar spacing equation (equation 1) derived from Bragg’s law (2d sin θ = λ, where n = 1):


$$\begin{array}{c} \frac{\text{1}}{{\text{d}}^{\text{2}}}=\;\left(\frac{\text{4}}{\text{3}}\right)\times \;\left[\frac{\left({\text{h}}^{\text{2}}+\text{ hk }+\;{\text{k}}^{\text{2}}\right)}{{\text{a}}^{\text{2}}}\right]+\;\left(\frac{{\text{l}}^{\text{2}}}{{\text{c}}^{\text{2}}}\right) \end{array}$$ (1)

where ‘d’ is the interplanar spacing, (h, k, l) are the Miller indices of the crystallographic planes, and ‘a’ and ‘c’ are the lattice constants.

This yielded a = 3.243 Å and c = 5.195 Å, with a c/a ratio of 1.602, closely matching the standard wurtzite ZnO structure (a = 3.249 Å, c = 5.206 Å). Such consistency indicates structural integrity and the absence of secondary phases, suggesting a high-purity crystalline material, as displayed in equation (1). The reference intensity ratio (RIR) is 5.64, indicating a moderate scattering profile in multiphase analysis. This structural data supports the assignment of ZnO to the ZnS(2H) polytype and forms the basis for its widespread use in optoelectronic, photocatalytic, and nanocomposite applications [44].

To explicitly distinguish the structural lattice parameter calculations from particle size determination, the crystallite size (D) was independently calculated from the most dominant peak, the (101) reflection at 2θ = 36.33°, using the Scherrer equation (equation 2).


$$\begin{array}{c} D=\frac{K\lambda }{\beta \;Cos\;\theta^{\prime }} \end{array}$$ (2)

Where K = 0.9 is the shape factor, λ = 0.15406 nm, Cu Kα wavelength, β = 0.25° = 0.00436 rad is the FWHM, and θ = 18.167° is the Bragg angle. The calculation gives


$$\begin{array}{c} D\;=\;\frac{\text{0}.\text{9}\;X\;\text{0}.\text{1}\text{5}\text{4}}{\text{0}.\text{0}\text{0}\text{4}\text{3}\text{6}\;X\;\text{0}.\text{9}\text{4}\text{9}}\;=\;\text{3}\text{3}.\text{5}\;nm \end{array}$$

Furthermore, the average crystallite size calculated from the observed diffraction peaks yields an average value of 36.1 nm (SD6a).

The Williamson–Hall approach further quantifies the lattice strain (ε) using equation 3 below


$$\begin{array}{c} \beta \;cos\;\theta \;=\frac{K\lambda }{D}+\text{4}\varepsilon \;sin\;\theta \end{array}$$ (3)

By substitution ε = 1.36 x 10^−4^, which signifies a negligible degree of lattice distortion, consistent with highly crystalline nanostructures. The low strain and narrow peak profiles reflect the uniformity of particle size and minimal defect density in the ZnO lattice.

Hence, XRD characterization of the synthesized ZnO nanocrystals confirmed a pure hexagonal wurtzite phase with refined lattice constants a = 3.243 A˚ and c = 5.195 A˚, an average crystallite size of 36.1 nm, and a microstrain of 1.4 × 10^−4^. The structural analysis confirms the formation of high-quality ZnO nanocrystals with excellent crystallinity and potential for wax-inhibition applications.

### FESEM Analysis of synthesized ZnO NPs

The Field Emission Scanning Electron Microscopy (FESEM) analysis of the biosynthesized ZnO NPs provides critical insights into their surface morphology, particle size, and microstructural characteristics (Fig 6a, SD6). The FESEM micrograph revealed predominantly spherical to hexagonal-shaped of ZnO NPs, a morphology consistent with green synthesis methods employing plant extracts, as also reported by Deepk et al. [45].

**Fig 6:**
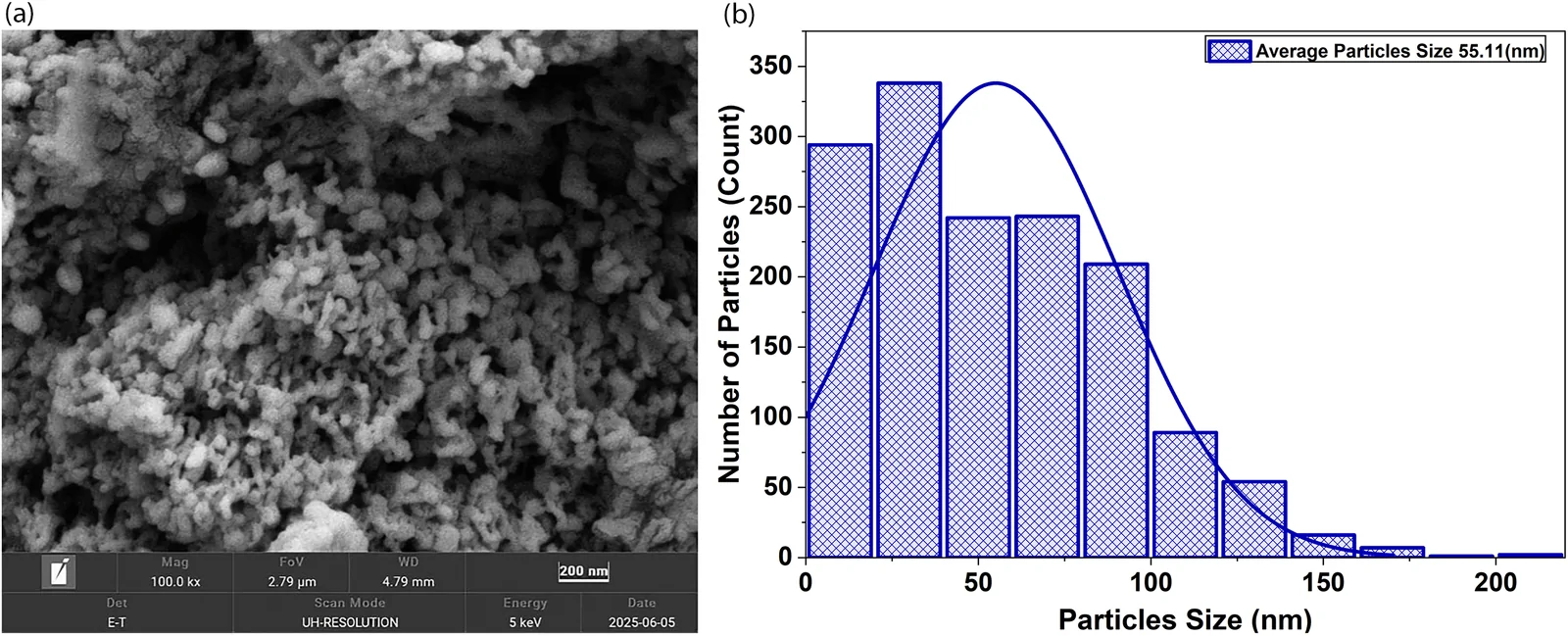
FESEM morphology and particle size distribution of bio-mediated ZnO NPs. (a) A micrograph revealing different sized NP (b) The particle size distribution histogram.

A different region of the FESEM micrograph was also examined, where particle sizes below 100 nm were recorded, specifically 32.03 nm, 51.23 nm, 51.76 nm, and 90.64 nm. XRD confirmed the presence of as low as 33.4 nm sized NP. The particles exhibit uniformity and distinct boundaries, indicating successful nucleation and growth under bio-mediated synthesis conditions [46].

The histogram of particle size distribution (n = 1,495), shown in Fig 6b (SD6), provides a more comprehensive statistical representation, revealing a mean (average) diameter of 55.11 nm with a positively skewed distribution. The modal population is concentrated within the 20–60 nm range, aligning well with the discrete FESEM measurements and the result obtained using the Scherrer relation (equation 2). The extended tail toward larger sizes (~100–200 nm) indicates agglomeration or secondary growth.

The FESEM-EDS profile as indicated in Fig 7a (SD7), substantiates the morphological consistency and elemental purity of the green-synthesized ZnO NPs. The Zn-rich phase (64.3 wt%) detected by EDS confirms successful metal oxide formation, while the residual carbon (15.1 wt%) and oxygen (20.6 wt%), as displayed in Fig 7b (SD7), indicates the presence of organic stabilizers and oxide framework, respectively, as supported by the literature [47]. The surface morphology appears slightly porous, suggesting residual organic capping or incomplete crystallization. This porosity and the presence of organic residues are characteristic of green synthesis routes and may enhance the NPs’ surface area and reactivity.

**Fig 7:**
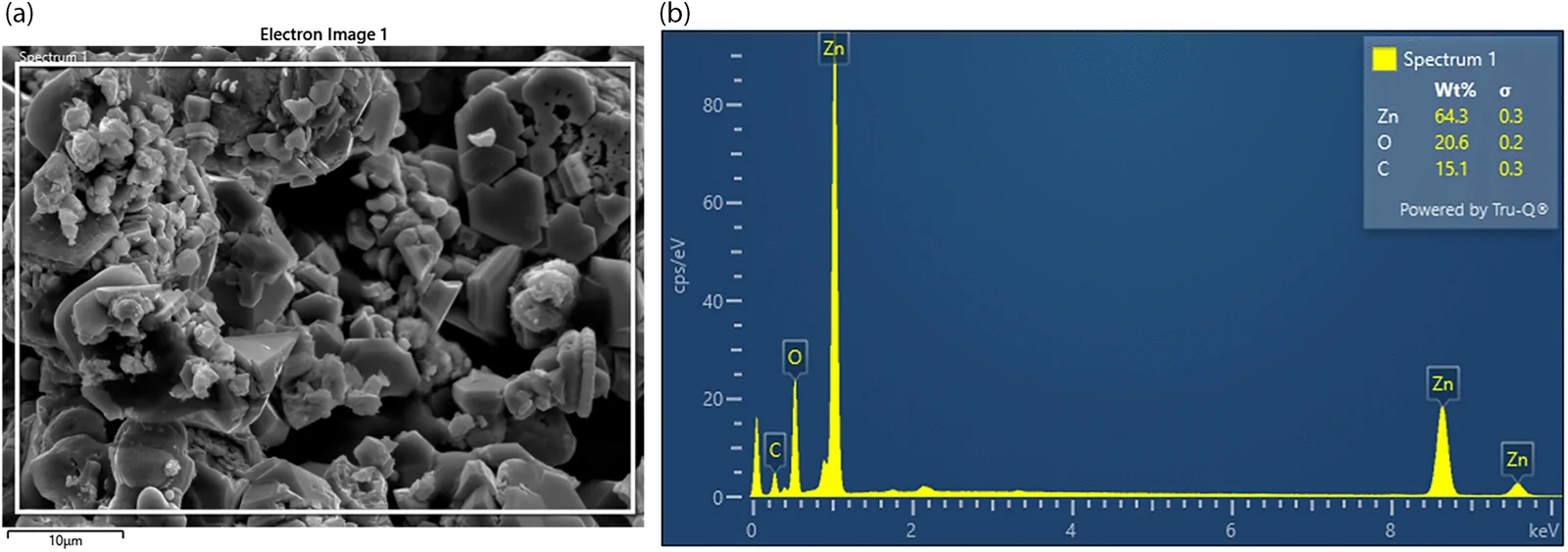
FESEM-EDS profile of ZnO. (a) FESEM micrograph showing aggregated ZnO grains with porous surface features. (b) EDS spectrum confirming Zn-rich phase.

Previous studies have demonstrated that materials with high surface area and surface-active functionalities play a critical role in modifying wax crystallization and deposition. For instance, Silica and alumina NPs have been shown to effectively reduce the wax appearance temperature and modify wax crystal growth in oil systems. These NPs act as heterogeneous nucleation sites, leading to a delayed onset of wax crystallization, reduced wax deposition, and altered crystal morphology, typically resulting in smaller, more regularly shaped, and finely dispersed wax crystals [48].

The surface-bound carbon may contribute to steric hindrance and functional group diversity, enhancing np dispersibility and interfacial activity in hydrocarbon matrices [49,50], making such NPs highly effective for applications ranging from biomedical imaging to catalysis and emulsion stabilization [51].

The EDS mappings (Fig 8, SD8) from this dataset further confirm the spatial homogeneity and compositional depth of the green-synthesized ZnO NPs. Clear, distinct distributions of Zn (purple), C (red), O (green), and N (orange) throughout the scanned area indicate well-integrated elemental domains. The presence of oxygen is directly associated with the formation of the ZnO lattice, thereby confirming the presence of the oxide framework. The nitrogen signal, albeit faint, is particularly noteworthy, as it implies incorporation of nitrogenous organic compounds from the bio-precursor, potentially offering additional surface functionalities [52] or from the substrate -NO_3_ used during the synthesis. This indeed contributes to the steric and electronic modulation at the particle surface. Such features can enhance binding with hydrocarbon chains and promote thermal dissipation; qualities essential for delaying wax nucleation in crude oil systems [53].

**Fig 8:**
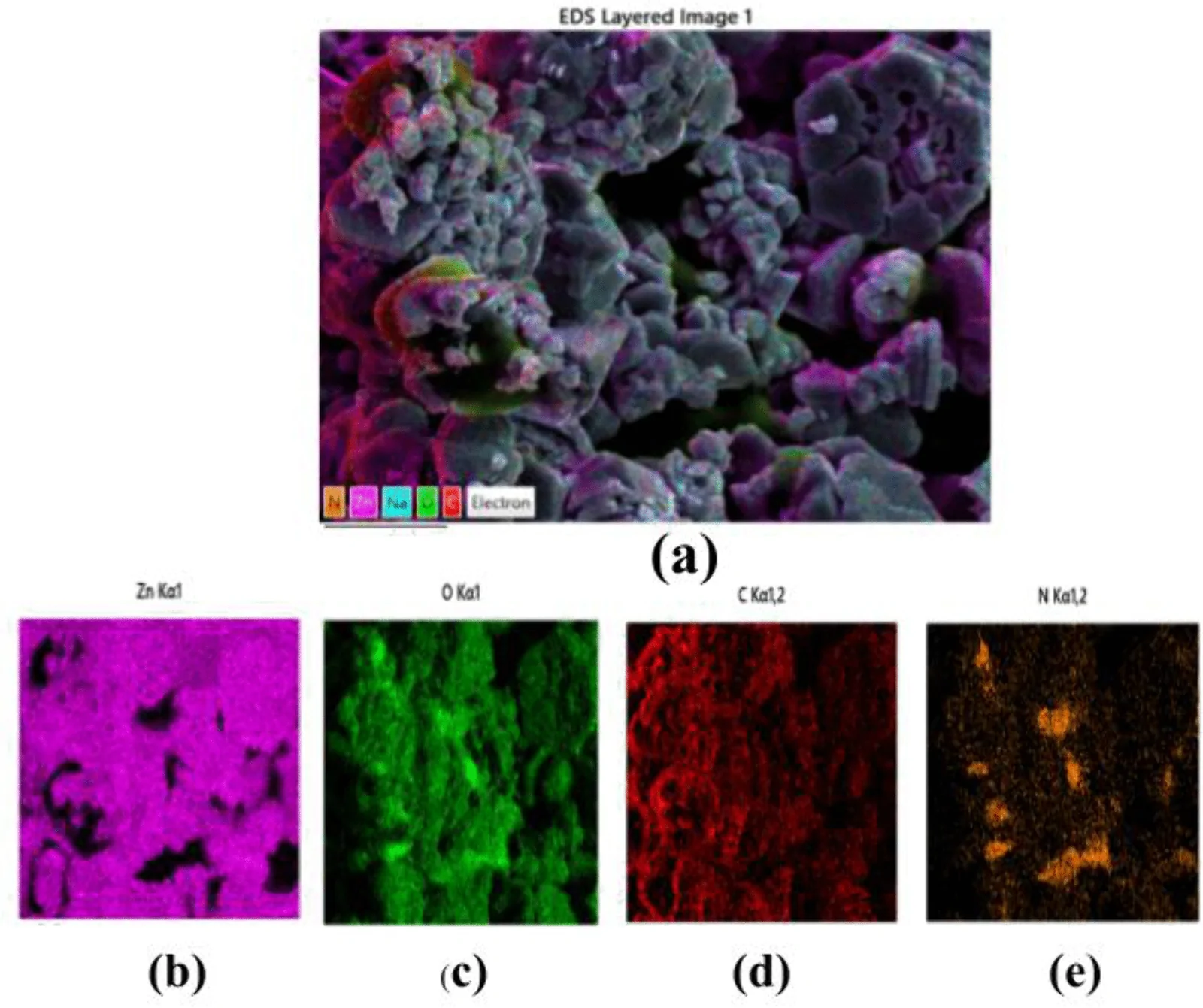
EDS elemental mapping and analysis of bio-mediated ZnO NPs. (a) Layered EDS image, (b) Zn distribution, (c) O distribution, (d) C distribution, and (e) N distribution.

Therefore, the multifunctionality underscores the dual role of green synthesis; generating the ZnO oxide matrix and simultaneously introducing organic residues that tailor interfacial behavior in crude oil systems.

### TGA Profile of ZnO NPs

TGA of the biosynthesized ZnO NPs reveals a stable thermal profile. This can be seen in Fig 9 (SD9) below. The weight change observed over the temperature range from ~30 °C to ~800 °C is minimal, with three distinct mass loss events recorded.

**Fig 9:**
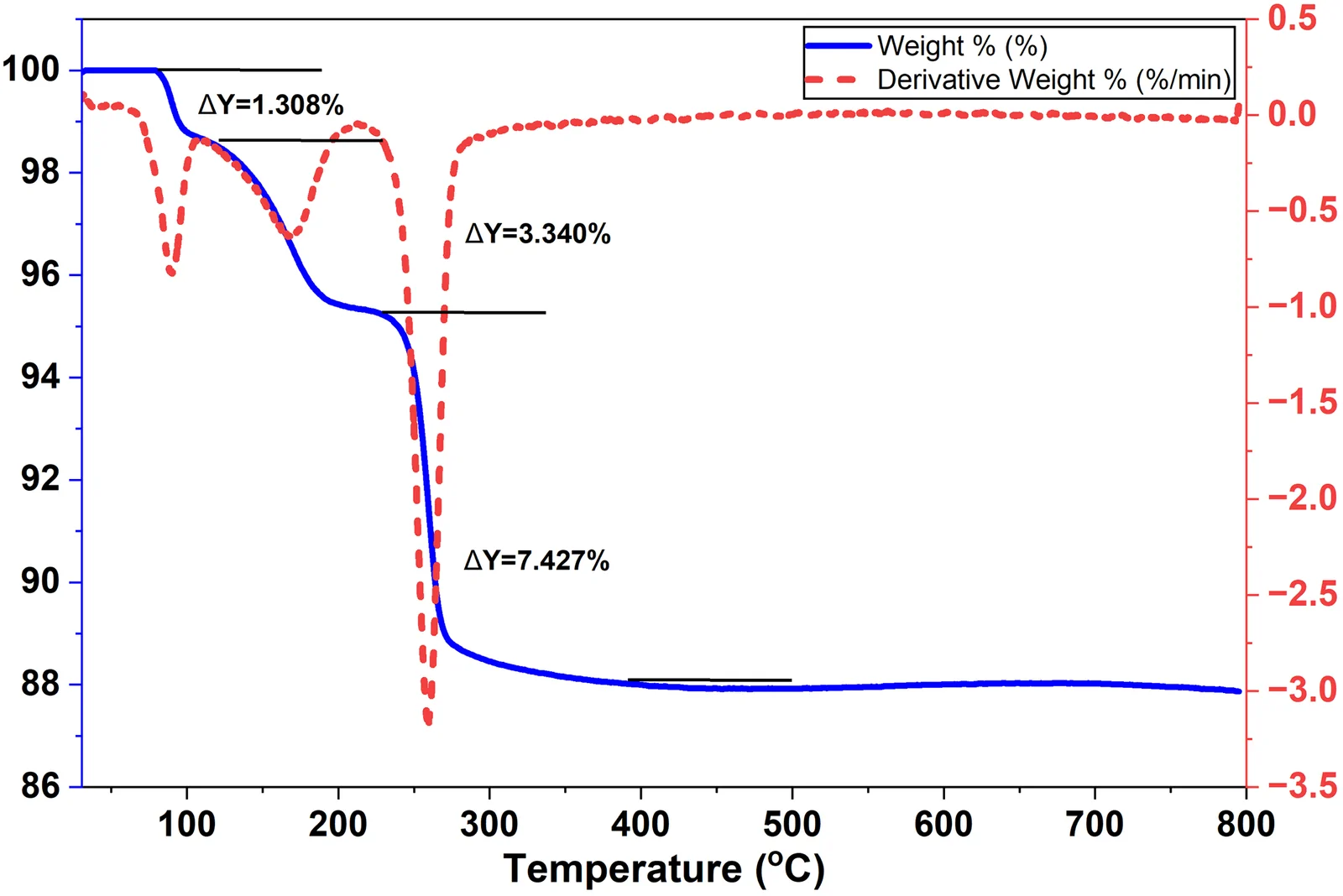
TGA analyses of *V. doniana* assisted synthesized ZnO Np.

In the first weight loss (80.00 °C to 118.71 °C, ΔY = 1.308%), the minor decrease corresponds to the desorption of physically adsorbed moisture and volatile organic remnants from the green synthesis using *V. doniana* extract, confirming the presence of biodegradable stabilizers and effective post-synthesis drying [69].

The second weight loss (118.71 °C to 227.90 °C, ΔY = 3.34%) can be ascribed to the partial decomposition of surface-bound hydroxyl groups, adsorbed oxygen species, and residual phytochemical capping agents. The relatively small magnitude of this loss indicates limited organic content and reinforces the purity and thermal resilience of the ZnO phase [51]. Between 227.90 °C and 391.77 °C, the sharp weight loss (ΔY = 7.427%) can be attributed to the release of trapped gases and residual organics from the inner pores, which is consistent with the restructuring of the porous ZnO network [70] and likely to the decomposition of firmly bound carbonaceous residues or by-products that survived the initial temperature [71].

These results indicate that the observed mass variations after calcination do not signify incomplete thermal treatment but rather reflect intrinsic porosity, surface activity, and the higher sensitivity of TGA to detecting minor thermal events compared to bulk furnace calcination, which aligns with prior studies on ZnO NPs thermodynamics [72]. Hence, limited total mass loss (<12% up to ~800 °C) demonstrates that the biosynthesized ZnO NPs are predominantly inorganic, with only a modest fraction of bio-derived stabilizers.

### ZnO inhibition capacity: Rheological measurement

Rheological measurements, specifically Temperature Sweep tests, were conducted using a rheometer to determine the effect of ZnO inhibitors on the flow characteristics of Dulang crude oil (Fig 10, SD10).

**Fig 10:**
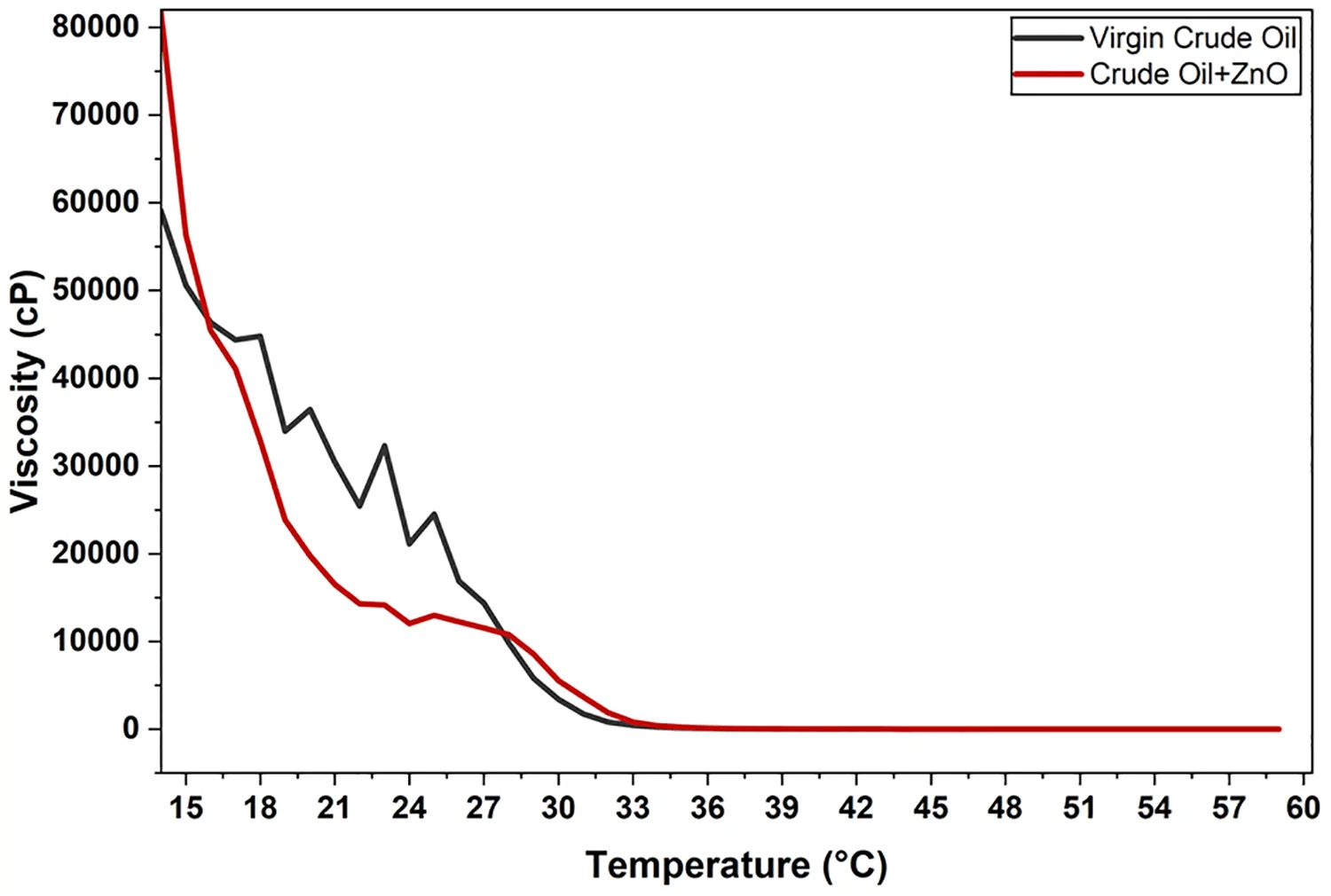
Temperature sweep rheological analysis of Dulang crude oil with ZnO inhibitors.

The Temperature Dependent Viscosity (TD-VR) index is calculated using equation 4 below.


$$\begin{array}{c} TD-VR\;(T)\;=\;\frac{\mu \text{0}(T)\;-\;\mu t(T)}{\mu \text{0}(T)} \end{array}$$ (4)

While the Viscosity Reduction is calculated by equation 5 below


$$\begin{array}{c} PIE\;(\%)=\frac{\mu Virgin(T)\;-\;\mu treated(T)}{\mu Virgin(T)}\times 100 \end{array}$$ (5)

Where:

μVirgin Viscosity of untreated crude at temperature T

μtreated Viscosity of treated crude at temperature T

The viscosity-temperature profile demonstrates the thermal response of virgin Dulang crude oil compared to the same crude oil modified with ZnO NPs. At the initial temperature range (15–20 °C), the virgin crude oil exhibits a significantly lower viscosity, approximately 60,000 cP, indicating the dominance of a rigid wax-gel structure characteristic of waxy crude oil. However, the ZnO-modified crude oil displays a markedly higher viscosity within this same temperature window, initially acting as obstacles before inhibiting crystal growth. Between 20 °C and 28 °C, the viscosity of both fluids decreases sharply, but the reduction is more pronounced in the treated crude oil, reflecting improved thermal sensitivity and a lower resistance to shear. This is consistent with the hypothesis that ZnO NPs act as nucleating agents or steric modifiers, altering the size, aggregation behaviour, and growth patterns of paraffin crystals. Consequently, the treated crude reaches a semi-fluid state earlier than the virgin crude oil. Beyond 30 °C, both viscosity curves converge to values below 1,000 cP, indicating the melting or dissolution of wax structures and the transition to a fully flowable liquid phase. The near overlap of the two curves at elevated temperatures also indicates that NP modification primarily influences low-temperature rheology rather than high-temperature behaviour, where viscosity is governed predominantly by the intrinsic composition of the lighter and intermediate crude oil fractions [54].

Hence, the results confirm that nanoparticle-assisted modification enhances mobility, reduces energy demand for pumping, and mitigates the risk of wax deposition under cold-start and sub-ambient temperature conditions [55].

The temperature dependence of the percentage inhibition efficiency (PIE) for the ZnO-treated virgin crude oil (VCO) reveals a clearly non-monotonic inhibition behaviour (Fig 11, SD11). This reflects the complex interplay between wax crystallization thermodynamics and nanoparticle-wax interactions. At lower temperatures (16–17 °C), the PIE values are minimal (≈1.9–7.4%), indicating that ZnO exhibits limited effectiveness when wax crystallization is already well established and dominated by strong crystal–crystal interactions.

**Fig 11:**
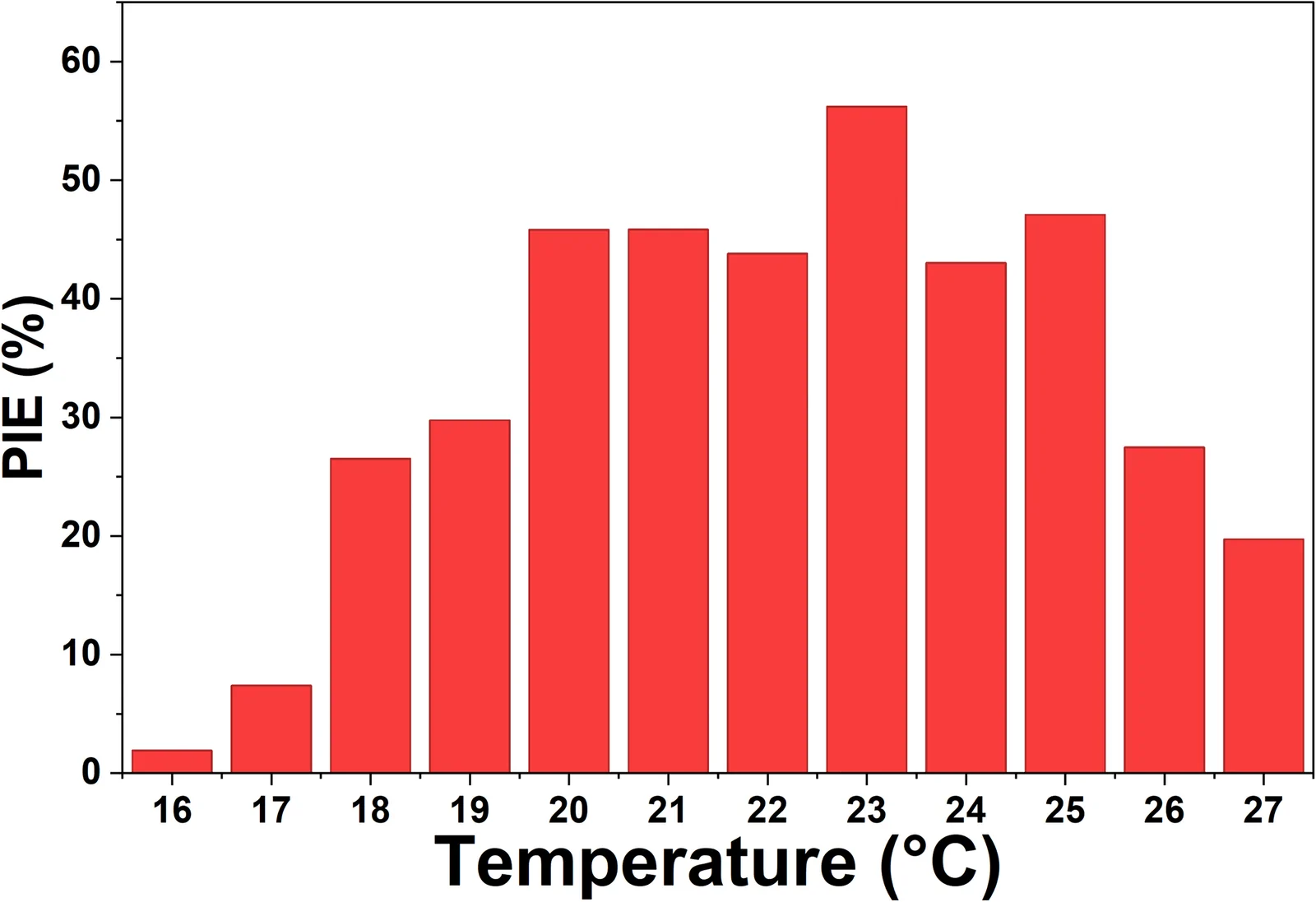
Temperature-dependent PIE of bio-mediated ZnO nanoparticles against paraffin wax crystallization.

As the temperature increases from 18 to 23 °C, a pronounced enhancement in inhibition efficiency is observed, with PIE rising sharply to approximately 56.2% at 23 °C. This temperature window corresponds to the vicinity of the wax appearance and early crystallization region, where wax crystals are nascent, smaller, and more susceptible to surface adsorption and steric interference by ZnO NPs. The high PIE in this range suggests that ZnO effectively adsorbs onto active growth sites of wax crystals, suppressing crystal growth, reducing interlocking, and weakening the three-dimensional wax network responsible for viscosity build-up.

Beyond the optimal region (24–27 °C), inhibition efficiency declines progressively, falling to approximately 19.7% at 27 °C. This reduction can be attributed to the diminished extent of wax crystallization at higher temperatures, where fewer solid wax structures are present for the inhibitor to interact with. Consequently, the apparent inhibitory effect decreases, not because of reduced NP performance, but because the baseline wax content in the solid phase is lower. Additionally, partial NP agglomeration at elevated temperatures may reduce the effective surface area available for wax-particle interactions.

The results demonstrate ZnO nanoparticles reduced crude oil viscosity by 56.2% at 23 °C. This means easier pumping and reduced wax deposition risk, which is in line with the study conducted, as demonstrated in Table 5 (T5) below:

**Table 5:** Comparative performance of nanoparticles as wax inhibitors in crude oil.

Material	Max viscosity reduction	Concentration	Reference
Fe_3_O_4_–NiO	42.6%	0.5 wt%	[56]
TiO_2_, MgO, Al_2_O_3_ NPs	TiO_2_ 27.9%, Al_2_O_3_ 23.5%, MgO 22.6%	2000 ppm	[57]
*V. doniana* assisted synthesised ZnO Np	56.2%	0.04 wt %	This work

The comparative results highlight the superior performance of *V. doniana*–assisted ZnO nanoparticles, which achieved a viscosity reduction of 56.2% at only 0.04 wt%, outperforming other reported nanoparticle systems such as TiO_2_, Fe_3_O_4_–NiO, and MgO/Al_2_O_3_. This demonstrates that biogenic ZnO nanoparticles synthesized via green methods can deliver enhanced inhibition efficiency at significantly lower concentrations, underscoring their potential as sustainable and cost-effective alternatives for crude oil flow assurance.

### Cross-polarized microscopy analyses

The CPM test was utilized to investigate wax crystal precipitation in Dulang crude oil samples treated with ZnO. Samples were subjected to a constant cooling rate over 70 minutes, during which the temperature was reduced, and the wax appearance and morphology, as well as the interaction between the wax and the ZnO inhibitor, were monitored. Fig 12 (SD12) displays representative images captured at the initial Wax Appearance Temperature (WAT) for the untreated (Virgin) Dulang crude oil and the Dulang crude oil treated with 0.04% ZnO, respectively.

**Fig 12:**
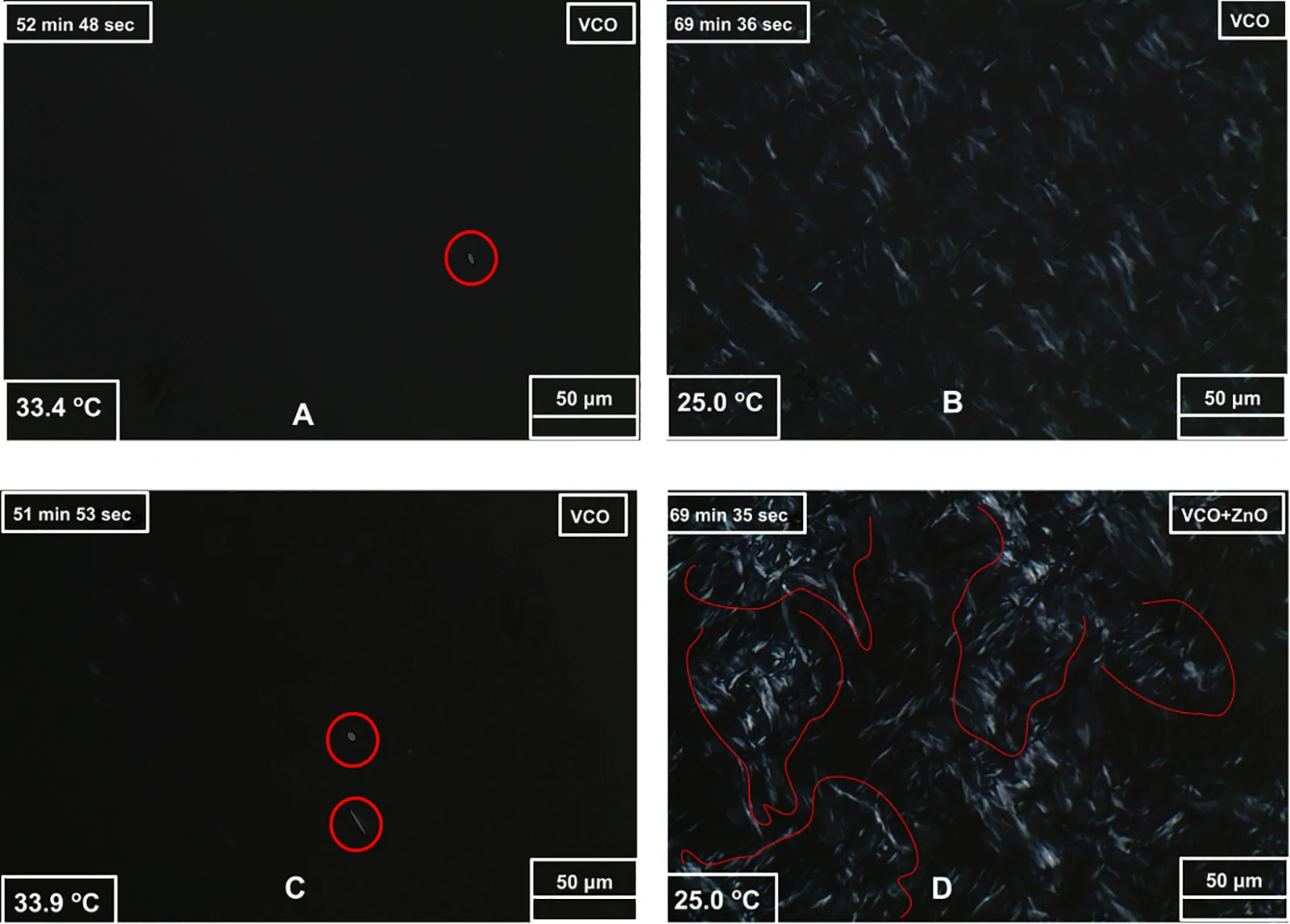
Comparative wax crystallization behavior in Dulang crude oil. (a) First Wax Appearance for Dulang VCO (b) Wax Precipitation at the end of experiment for VCO (c) First wax appearance for Dulang crude oil + 0.04 wt% ZnO (d) Wax Precipitation at the end of experiment Dulang Crude oil + 0.04 wt% ZnO.

Based on the observed wax crystalline morphology, the precipitated wax was primarily characterized as macrocrystalline. This observation is consistent with reported literature, which suggests that upon temperature reduction, macrocrystalline waxes typically aggregate to form needle-shaped or flat plate-like crystals [58]. For the untreated crude oil (highlighted in the red boxes in Fig 12a), the initial appearance of faint white wax formations (WAT) was observed at 33.4 ˚C. In contrast, Fig 12b depicts more pronounced needle-shaped wax formations following further temperature reduction.

For the crude oil treated with ZnO (highlighted within the red circle in Fig 12c), the initial appearance of faint white wax formations (WAT) was observed at a higher temperature of 33.9 ˚C. Conversely, Fig 12d demonstrates more pronounced needle-shaped wax formations after the temperature was decreased; however, the wax appeared in a clustered morphology. This observation suggests that the inhibitor’s mechanism of action involves altering the wax structure through cocrystallization (indicated by the red line margin in Fig 12d). Hence, the inhibitor increased the WAT by 0.5 ˚C and modified the wax network by inducing crystallization into nuclei. This structural alteration may account for the observed reduction in activation energy, and as the temperature continues to decrease, the intrinsic rheology of the modified wax further inhibits flow resistance [59].

### Flow activation energy

The temperature dependence of viscosity was analyzed using the Arrhenius relationship (equation 6) by plotting ln(μ) against inverse temperature (1/T). A strong linear correlation, R^2^ > 0.92, was obtained for both virgin crude oil and ZnO-treated samples (Fig 13, SD13). The calculated flow activation energy decreased from 213.6 kJ mol ^−^ ¹ for the virgin crude oil to 207.6 kJ mol ^−^ ¹ after ZnO addition, indicating a reduction in the energy barrier for flow. This confirms the effectiveness of ZnO NPs in improving crude oil flow behaviour.


$$\begin{array}{c} {Ea}_{VCO\;or\;Co+ZnO}=m\;X\;R \end{array}$$ (6)

**Fig 13:**
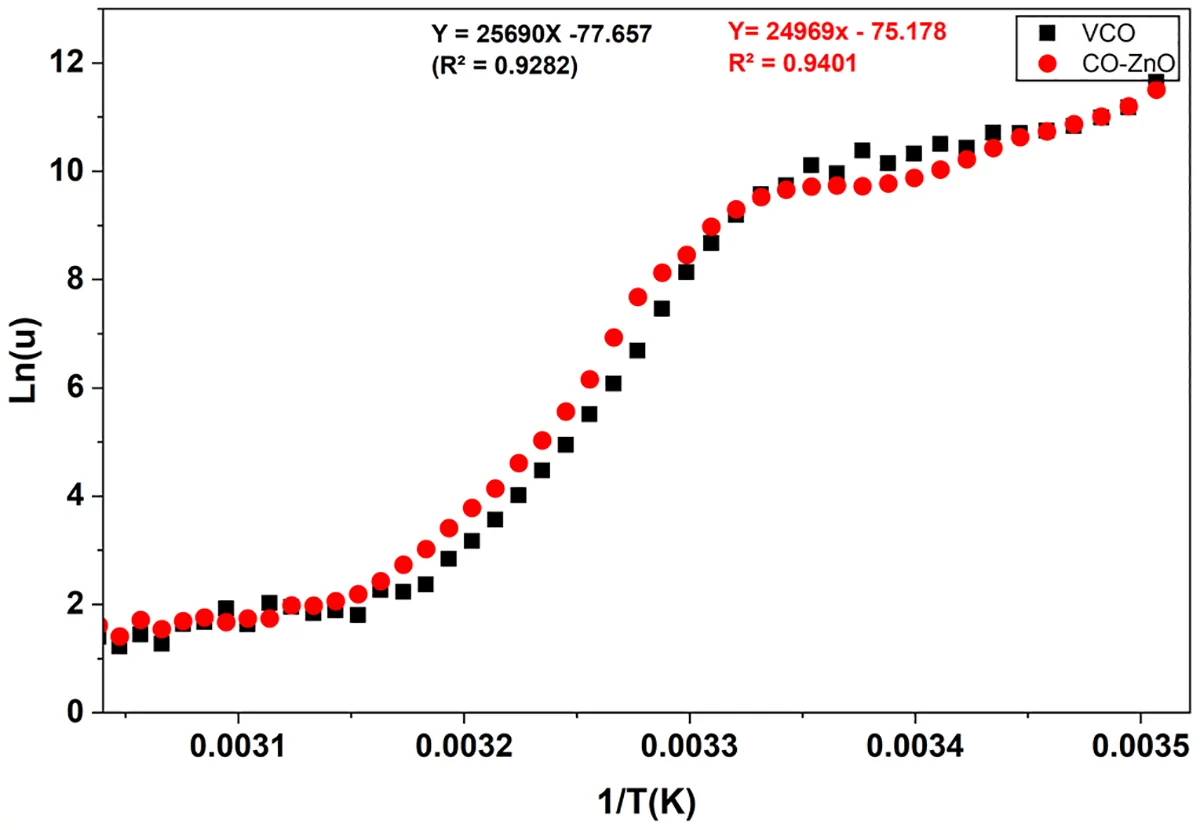
Flow activation energy analysis of Dulang crude oil with and without ZnO nanoparticles.

**Table:** 

Activation Energy for Virgin Crude Oil Ea_VCO_ = m × R	Activation Energy for Crude Oil + ZnO Ea_CO + ZnO_ = m × R
Where:	Where:
m = 25690	m = 24969
R = 8.314 J mol ^−^ ¹ K ^−^ ¹	R = 8.314 J mol ^−^ ¹ K ^−^ ¹
Ea = 25690 X 8.314 = 213,586.7 J mol ^−^ ^1^	Ea = 24969 X 8.314 = 207,592.3 J mol^−1^
213.6 kJ mol ^−^ ^1^	207.6 kJ mol ^−^ ^1^

### Reduction in Activation Energy is


$$\begin{array}{c} \Delta \text{Ea }=\text{ 213}.\text{6}\;-\text{ 207}.\text{6}\;=\;\text{6}.\text{0}\text{ kJ mo}l^{-\text{1}} \end{array}$$

The incorporation of ZnO NPs reduced the flow activation energy of the crude oil from 213.6 to 207.6 kJ mol ^−^ ¹, indicating a lower energy barrier for molecular mobility and flow. This reduction confirms the role of ZnO as an effective wax inhibitor by weakening intermolecular associations and disrupting wax-crystal networks, thereby enhancing flowability [60].

## Conclusion

This study establishes a sustainable and functionally effective pathway for wax mitigation via the green synthesis of ZnO nanoparticles using *V. doniana* leaf extract. The novelty of this work is twofold. For the first time, this study demonstrates the successful use of V. doniana phytochemicals as bio-reductants and stabilizing agents in the synthesis of phase-pure ZnO nanoparticles. The resulting particles exhibit circular, irregular, and hexagonal wurtzite structures (a = 3.243 Å, c = 5.195 Å), nanoscale crystallite size (~33.5 nm with mean particle size of 36.1 nm), and low lattice strain (1.4 × 10 ^−^ ⁴), confirming their high crystallinity and structural integrity. Second, it provides the first systematic evaluation of these bio-derived ZnO nanoparticles as wax inhibitors in a real waxy crude oil system.

The synthesized nanoparticles exhibited favorable physicochemical properties, high thermal stability (total mass loss < 12.1% up to ~800 °C), and surface functionalization by residual phytochemicals, collectively enhancing interfacial activity. These attributes translated into measurable improvements in flow assurance, with a maximum viscosity reduction of 56.2% at 23 °C, a decrease in flow activation energy from 213.6 to 207.6 kJ mol ^−^ ¹, and a peak inhibition efficiency of ~56.2% within the wax crystallization window. Complementary cross-polarized microscopy confirmed a transition from needle-like wax structures to clustered morphologies, evidencing a cocrystallization-based inhibition mechanism.

Despite these promising findings, certain limitations should be acknowledged. The study was conducted at a single nanoparticle concentration (0.04 wt%), and thus does not capture concentration-dependent optimization or threshold behavior. Additionally, the experiments were performed under controlled laboratory conditions using a single crude oil type (Dulang crude), which may limit direct generalization to field-scale pipeline systems with varying compositions, pressures, and shear environments. Furthermore, long-term dispersion stability, potential nanoparticle agglomeration under dynamic flow conditions, and environmental impact assessments were not addressed and warrant further investigation.

Therefore, the findings provide strong experimental evidence that plant-mediated ZnO nanoparticles can serve as environmentally benign and effective wax inhibitors, while also highlighting the need for extended parametric and field-scale validation to fully establish their industrial applicability.
